# Annotations

**Published:** 1911-04-15

**Authors:** 


### Annotations.

### The Overstocking of the Profession in France.

There would seem to be a reaction, at any rate in France, in favour of compulsory training in classics prior to admission to the ranks of students in medicine. At the last meeting of the Association de la presse médicale française, the *limitation in number of doctors* was discussed, a question brought forward by the Vigilance Committee of the Congress of Practitioners. It was decided unanimously that such limitation was impracticable and undesirable, but the meeting held that the course of preliminary studies should be very thorough, and should include classics. Dr. Blondel next drew attention to a matter which was of great importance to the native practitioners. Owing to exemptions which the holding of a degree in science or modern languages procured, many foreigners entered into competition with Frenchmen. Such degrees were easy to obtain, especially by foreigners, in the case of modern languages. The meeting decided to draw the attention of the French Senate to the matter, and a committee was chosen, to which powers were given to act so that the decree might be rescinded whereby exemptions were granted to holders of the degrees in question.

### Flour and Public Health.

The interesting report on the bleaching of flour just issued by the Local Government Board, and which we summarise on p. 73 of this issue, comes at an appropriate moment, for during the last six months the public has been greatly exercised in mind with regard to the merits of certain qualities of flour. The conclusions reached in the report are not by any means new, but they serve to draw attention to the harmful consequences that may follow attempts to promote the "look" of foodstuffs by adulterating them with chemicals which, although not poisonous in the strict sense of the term, are noxious things in so far that they materially damage the organism. The recommendations with regard to legislative provision for the prevention of flour-adulteration will meet with general approval. At the same time, it is wise to insist that colour alone is not a criterion of the quality of bread. It has often been pointed out that coarse breads and clay-coloured flours lend themselves much more easily to "artistic adulteration" than the white sorts, and are less easily judged by the uninitiated. Anyone with a wholesome sense of taste can detect the admixture of harmful adulterants in a white loaf, but few can tell, by mere tasting, the presence of chalk in brown bread. Superiority is a question of quality, and in judging breads it is necessary to take many other things into consideration besides colour.

### The Modern Treatment of Fracture.

The special Commission appointed by the British Medical Association to investigate the subject of the surgical treatment of fractures will have to decide some very important points, and it is no exaggeration to say that the whole profession in this country waits its report with interest. The work done abroad by Bardenheur, and Codavilla, and in this country by Mr. Arbuthnot Lane among others, has shown that the results obtained in fracture cases from what appear to be entirely opposing lines of treatment are such as to give thorough satisfaction to those who favour one or other line of treatment. We have recently (The Hospital, January 7, 14, 21, 28) given a *resumé* of Mr. Lane's teaching and a brief description of the technique he adopts: we hope in an early issue to outline in a similar manner the teaching and procedure of the Cologne school, in order to give our readers an opportunity to compare and contrast the two methods. There is no doubt that the whole subject of fractures and their treatment demands impartial and thorough investigation, and we trust that the Commission will give it such consideration and publish the fullest details about its investigations at an early date. The data upon the subject are so diffused and scattered that the ordinary practitioner has no means of judging between the claims of the two schools.

### Duelling and the Profession.

The Medical Chamber of Galicia (Austria) has recently been called upon to give judgment in a somewhat peculiar case. An unhappy widow whose son had been killed in a duel lodged a complaint against a doctor who had waited upon the combatants, maintaining that he had committed a breach of duty not only by being present at the duel, but also by not warning in advance the authorities and the parents of the duellists. The Chamber expressed the opinion that there would be a real danger in leaving combatants without professional help in case of grave injury; and that, in addition, the doctor, who has been told in confidence that a duel is to take place, must on no account betray the secret. An international inquiry on the following points is to be opened on account of this case. (1) Does a doctor commit a breach of duty in agreeing to be present at a duel? (2) In the event of it being proper for a medical man to assist, must he always do so, or only in certain circumstances? (3) On the other hand, must he always refuse, or only under certain circumstances? (4) When a doctor is warned of a duel must he intervene to prevent it? (5) Is it desirable that the law should be modified so as to allow of doctors acting with impunity in such cases instead of, as now, being treated on the same terms as ordinary witnesses? (6) If cases should arise in which the presence of a doctor is questionable, ought he to be obliged to consult a colleague on the point? (7) Is it not desirable, in view of the fact that both combatants may be grievously injured, that two doctors should be present at a duel? (8) Ought not the point to be discussed by the various professional associations? (9) Have you any remarks to make based on your own professional experience? Answers are to be sent to M. Mikolajski, 6 Rue Sniadeckich, Lwow (Austria).

